# A template-based approach for responsibility management in executable business processes

**DOI:** 10.1080/17517575.2017.1390166

**Published:** 2017-10-27

**Authors:** Cristina Cabanillas, Manuel Resinas, Antonio Ruiz-Cortés

**Affiliations:** ^a^ Institute for Information Business, Vienna University of Economics and Business, Vienna, Austria; ^b^ Depto. Lenguajes y Sistemas Informáticos, University of Seville, Seville, Spain

**Keywords:** Business process management, BPMN, RAL, resource assignment matrix, responsibility management

## Abstract

Process-oriented organisations need to manage the different types of responsibilities their employees may have w.r.t. the activities involved in their business processes. Despite several approaches provide support for responsibility modelling, in current Business Process Management Systems (BPMS) the only responsibility considered at runtime is the one related to performing the work required for activity completion. Others like accountability or consultation must be implemented by manually adding activities in the executable process model, which is time-consuming and error-prone. In this paper, we address this limitation by enabling current BPMS to execute processes in which people with different responsibilities interact to complete the activities. We introduce a metamodel based on Responsibility Assignment Matrices (RAM) to model the responsibility assignment for each activity, and a flexible template-based mechanism that automatically transforms such information into BPMN elements, which can be interpreted and executed by a BPMS. Thus, our approach does not enforce any specific behaviour for the different responsibilities but new templates can be modelled to specify the interaction that best suits the activity requirements. Furthermore, libraries of templates can be created and reused in different processes. We provide a reference implementation and build a library of templates for a well-known set of responsibilities.

## 1. Introduction

Organisations need to manage the different types of responsibilities that their employees may have with respect to all the activities that are daily carried out within them. Process-oriented organisations need to do it, in addition, in accordance to the business processes in place. In this context, responsibilites are defined at different levels. As evidenced by several studies, there are four acknowledged process positions (business process director, business process consultant, business process architect and business process analyst) and a specific set of responsibilities associated to each of them (Antonucci and Goeke 2011). However, organisations need to control not only the execution of processes as a whole but also the execution of every single activity carried out within them, which relates to a key role in process execution: the process participants. Activities often require the collaboration among several people with different responsibilities, e.g., people responsible for performing the work, people acting as consultants who provide valuable input for the completion of the activity, and people accountable for the results. Therefore, there are also responsibilities at activity level involving, among others, accountability and consultation.

Business Process Management Systems (BPMSs) stand out of Process-Aware Information Systems (PAISs) as a mechanism for process automation. Specifically, the purpose of a BPMS is to coordinate an automated business process so that the work is done at the right time by the right resource (Dumas et al. 2013). They rely on the description of business processes as process models represented with different notations, such as (EPC) (Mendling, Neumann, and Nüttgens 2005) or the de-facto standard Business Process Model and Notation (BPMN) (OMG 2011). However, although BPMSs use business process models for automation, there is still a gap between these executable process models and business-oriented process models used for communication and analysis. Because of their intent, the latter are not as precise and complete as an executable process model must be (Dumas et al. 2013). As a consequence, different methodologies (Dumas et al. 2013) and techniques (Graml, Bracht, and Spies 2008; Caron and Vanthienen 2016) for transforming business-oriented process models into executable ones have been developed. Still, this transformation is known to be slow and error prone (Alotaibi and Liu 2017).

In this paper, we focus on this issue in the context of responsibility management. Nowadays, BPMSs are increasingly providing support for modelling activities that involve several people with different responsibilities using advanced resource assignment languages (Cabanillas et al. 2015b) or supplementary models like RACI matrices (Cabanillas, Resinas, and Ruiz-Cortés 2011b). However, this support tends to be limited to documentation and reporting purposes. Just a few BPMSs consider several responsibilities associated to an activity during process execution and the existing support suffers from generalisability and flexibility issues. Concepts like accountability or consultation, common in the domain of responsibility management, have to be implemented during the transformation from business-oriented process models to executable process models by manually adding activities for them. Furthermore, this has to be done for each activity that involves several responsibilities in all automated business processes in the organisation, which is time-consuming and error-prone as these transformation tasks usually are (Alotaibi and Liu 2017). Moreover, if the responsibilities defined for an activity change, the activities added manually to the process model may also change, which adds additional work that may be significant given the continuous organisational changes (Aldin and de Cesare 2011).

Two facts have contributed to the lack of advanced support for responsibility management. First, most process model notations used in current BPMSs for process execution support only one type of responsibility by default, despite some of them like BPMN allow including additional responsibilities in an ad-hoc fashion (OMG 2011). This lack of standardisation for managing various types of responsibilities discourages BPMS developers to support different responsibilities in their systems. Second, the way people with different responsibilities interact within an activity is domain-specific. At least, it depends on the organisation and the activity. For instance, some activities may require partial approvals of the work being performed for their completion, whereas for others, such an approval may only be required at the end of the execution or not required at all. Therefore, supporting different responsibilities is not only a matter of assigning new tasks to a worklist, but it is also necessary to find a mechanism that coordinates them in a flexible way.

In this paper, we automate this transition from busines-oriented to executable business processes in the context of responsibility management by enabling current BPMSs to execute processes in which people with different responsibilities interact to perform process activities. The approach involves two artifacts. On the one hand, we address the modelling of different responsibilities by extending a (RAM) (Website 2016) with information required for process execution. On the other hand, we introduce a template-based technique for transforming such information into BPMN elements that can be interpreted by a BPMS so that existing BPMN execution support suffices to automate process models that involve activities with several people with different responsibilities. This idea was previously described in Cabanillas, Resinas, and Ruiz-Cortés (2012) but the work has been extended in several directions, in particular: (i) our current approach does no longer enforce any specific behaviour for the people with different responsibilities that work together in an activity, but new templates can be modelled to specify the interaction that best suits the requirements of each activity. In fact, the whole template-based mechanism is new; (ii) our current approach is not limited to a restricted set of responsibilities anymore, hence gaining generalisability; and (iii) we have refined the previous metamodel and its semantics has been properly defined.

Our approach has two additional advantages. First, it is independent of the platform and hence, the models obtained can be used by any BPMS that supports BPMN . Second, the original structure of the process model remains unchanged after including the templates defined for modelling responsibilities, since the modifications are done at subprocess level. This provides transparency and does not affect the readability of the original model.

The paper is structured as follows. Section 2 describes a scenario that will be used throughout the paper as a running example. Section 3 summarises the related work. Section 4 motivates the work in line with the research method used. Section 5 presents our approach for modelling responsibility aspects in process activities. Section 6 introduces our template-based approach for generating resource-aware BPMN models. Section 7 describes the ways in which we have validated the approach. Section 8 reflects on advantages and limitations of the approach. Finally, Section 9 outlines the conclusions and directions for future work.

## 2. Running example

The following example is used to illustrate the importance of supporting the collaboration of several people with different responsibilities in a process activity.

**Figure 1.**
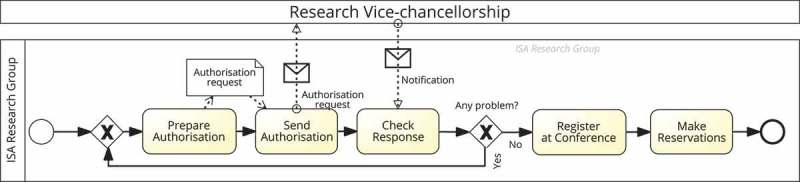
Business process to manage the trip to attend a conference.

Table 1 depicts an excerpt of an organisational model for a project called HRMS. Specifically, it shows the organisational roles assigned to the resources that contribute to the project.^1^ Figure 1 shows a simplified version of the procedure to manage the trip to a conference according to the rules of the University of Seville (Spain). In particular, it represents a *collaboration* between two processes modelled with BPMN 2.0^2^ (OMG 2011): one process is developed at pool *Research Vice-chancellorship* and the other at pool *ISA Research Group*, to which the organisational model previously described belongs. That process starts when a researcher requests for authorisation to attend the conference, for which an authorisation form is filled out with the details of the applicant and the funding source, and sent for external assessment to the Vice-chancellorship. After evaluation, a notification from the Vice-chancellorship is received informing about the approval or rejection of the request, which will be checked by *the researcher*. In the absence of problems, *the researcher* must register at the conference and make the reservations required.

**Table 1.** Excerpt of the organisational model for project HRMS (WP = Work Package).

	Project	Account	Resource	WP		
	Coordinator	Admin.	Manager	Leader	Researcher	Clerk
Anthony	✓		✓		✓	
Betty		✓				
Anna						✓
Charles				✓	✓	
Christine				✓	✓	
John					✓	
Adele					✓

The previous description of the process is only half of the picture because it assumes that only one person is involved in each activity, in this case a certain researcher. However, for most activities several people are actually involved in them with different responsibilities. For instance, the coordinator of the project that will finance the trip expenses is accountable for activity Prepare Authorisation and the clerk of the project helps the researcher in this task by providing the information required about the funding source; in addition, the account administrator and the leader of the project’s work package related to the subject of the paper to be presented in the conference (or the subject of the conference, in case of no accepted publications) must be informed about the trip request. Following up on this, the project coordinator and the account administrator are informed about the result of the request when executing activity Check Response. In activity Register at Conference, the project coordinator can be consulted about details on the registration process, such as the type of registration, and both the project coordinator and the account administrator must be informed after the registration has been done. Finally, in activity Make Reservations, the clerk of the project can help the researcher, if required, and the account administrator and the project coordinator can also be consulted about details on this activity.

The challenge is to model all those details and come up with a *responsibility-aware process model* that can be executed taking the responsibilities into account.

## 3. Related work

Responsibility management in business processes is a part of resource management in business processes, which involves the assignment of resources to process activities at design time as potential participants and the allocation of resources to activities at run time as actual participants.

Resource assignment languages (van der Aalst and ter Hofstede 2005; Cabanillas et al. 2015b; Bertino, Ferrari, and Atluri 1999; Strembeck and Mendling 2011; Casati et al. 1996; Scheer 2000; Du et al. 1999; Tan, Crampton, and Gunter 2004; Cabanillas et al. 2015a; Wolter and Schaad 2007; Awad et al. 2009; Stroppi, Chiotti, and Villarreal 2011) serve the former purpose by enabling the definition of the conditions that the members of an organisation must meet in order to be allowed to participate in the activities of the processes executed in it, e.g., to belong to a specific department or to have certain skills. The outcome is a *resource-aware process model*. The set of conditions that can be defined depicts the expressiveness of the language and is usually evaluated with a subset of the well-known workflow resource patterns (Russell et al. 2005), namely, the creation patterns, which include, among others: *Direct, Organisational, Role-Based*, and *Capability-Based Distribution*, or the ability to specify the identity, position, role or capabilities of the resource that will take part in a task, respectively; *(SoD)*, or the ability to specify that two tasks must be allocated to different resources in a given process instance; and *Retain Familiar* (also known as Binding of Duties *(BoD))*, or the ability to allocate an activity instance within a given process instance to the same resource that performed a preceding activity instance. A comparison of resource assignment languages can be found in Cabanillas et al. (2015b).

Resource allocation techniques aim at distributing actual work to appropriate resources so that process instances are completed properly, e.g, in terms of high quality and low time and cost (Havur et al. 2015). All process engines must be provided with some resource allocation mechanism(s) in order to automate process execution.

Traditional resource management in business processes considers that a process activity requires the workforce of one single resource who is in charge of the activity from the beginning to the end of its execution. However, common scenarios like the one described in Section 2 show the importance of other types of responsibilities, which tend to be disregarded by existing resource management approaches. In the following, we review the current state of the art on responsibility management in business processes, which is the problem addressed in this paper, and then report on approaches for process modelling based on templates, which relates to our solution.

### 3.1. Responsibility management in business processes

In this section, we first introduce a generic responsibility management mechanism that is independent of process modelling notations or BPMS . Afterwards, we explore the related work for responsibility management in business processes in three groups: (i) the support provided by existing process modelling notations, (ii) the support provided by current modelling software tools and BPMS, and (iii) research proposals developed to bridge existing gaps.

#### 3.1.1. Responsibility assignment matrices (RAMs)

A Responsibility Assignment Matrix (RAM) provides a way to plan, organise and coordinate work that consists of assigning different degrees of responsibility to the members of an organisation for each activity undertaken in it (Website 2016). RAMs were defined independently of Business Process Management (BPM) and thus, they are suitable for both process- and non process-oriented organisations. In the context of RAMs, the different responsibilities that may be assigned to an activity are usually called *roles* or *task duties* (Cabanillas et al. 2015b).

RAMs are becoming a recommendation for the representation of the distribution of work in organisations. As a matter of fact, a specific type of RAMs called RACI (ARIS 2012) is a component of Six Sigma,^3^ a methodology to improve the service or product that a company offers to its customers. There are also ongoing efforts to map RACI to the LEAN and CMMI for Services (CMMI-SVC) frameworks (Nuzen and Dayton 2011). The former defines a set of principles for continuous process improvement. The latter provides guidance for applying Capability Maturity Model Integration (CMMI) best practices in a service provider organisation. Similarly, the Information Technology Infrastructure Library (ITIL) framework defines the ITIL RACI matrices^4^ as the way to illustrate the participation of the ITIL roles in the ITIL processes. ITIL is the worldwide de-facto standard for service management. Specifically, it uses a modality of RAMs called RASCI (Website 2014), which relies on the following five responsibilities:

- *Responsible (R)*: person who must perform the work, responsible for the activity until the work is finished and approved by the person accountable for the activity. There is typically only one person responsible for an activity.
- *Accountable – also Approver or Final Approving Authority – (A)*: person who must approve the work performed by the person responsible for an activity, and who becomes responsible for it after approval. There is generally one person accountable for each activity.
- *Support (S)*: person who may assist in completing an activity by actively contributing in its execution, i.e., the person in charge can delegate work to her. In general, there may be several people assigned to this responsibility for an activity instance.
- *Consulted – sometimes Counsel – (C)*: person whose opinion is sought while performing the work, and with whom there is two-way communication. She helps to complete the activity in a passive way. In general, there may be several people assigned to this responsibility for an activity instance.
- *Informed (I)*: person who is kept up-to-date about the progress of an activity and/or the results of the work, and with whom there is just one-way communication. In general, there may be more than one person informed about an activity.

Table 2 illustrates an example of a RAM for the scenario described in Section 2, specifically a RASCI matrix. The rows represent the process activities, the columns of the matrix are organisational roles,^5^ and each cell contains zero or more RASCI initials indicating the responsibility of that role on that activity.

**Table 2.** RASCI matrix for the process at pool *ISA Research Group.*

	Project	Account	WP		
	Coordinator	Admin.	Leader	Researcher	Clerk
Prepare	A	I	I	R	C
Authorisation					
Send				R	
Authorisation					
Check	I	I		R	
Response					
Register at	C/I	I		R	
Conference					
Make Reservations	C	C		R	S
				

Note that RAMs are intended to be a responsibility modelling mechanism and are not provided with support for automated analysis that could help to use them together with business processes during process execution. Their expressive power is high in terms of the number of responsibilities that can be assigned but low regarding the number of workflow resource patterns supported, as constraints like sod and bod cannot be defined.

#### 3.1.2. Process modelling notations

The default support for responsibility management in current process modelling notations is limited. BPMN 2.0 (OMG 2011), the de-facto standard for process modelling, provides a mechanism to assign responsibilities to an activity. However, the only responsibility type that is defined by default is Responsible (so-called Potential Owner in BPMN). Other types of responsibilities can be added by extending the BPMN metamodel. In addition, nothing is said about the implications of adding new responsibilities during process execution.

The EPC notation (Dumas, van der Aalst, and ter Hofstede 2005) is more expressive than BPMN for resource modelling in the sense that it provides a specific representation of organisational units and allows defining organisational relations. However, to the best of our knowledge there is no support for responsibilities other than the resource in charge of executing the activity.

The so-called activity partitions of Unified Modeling Language (UML) Activity Diagrams (Russell et al. 2006) are classifiers similar to the BPMN swimlanes, although enriched with dimensions for hierarchical modelling. Therefore, they allow grouping process activities according to any criterion, which includes organisational information. Besides that, this modelling approach is very little expressive in terms of the support provided for the creation patterns (Russell et al. 2005). There is no notion of responsibility modelling either.

Finally, BPEL4People (OASIS 2009) is an extension of the BPEL notation (OASIS 2007) based on the WS-HumanTask specification (OASIS 2010), which enables the integration of human beings in service-oriented applications. It provides support for the execution of business processes with three types of responsibilities, namely: Responsible, Accountable and Informed. However, although it provides a rather flexible mechanism for defining the notifications that the people with responsibility Informed receive, the participation of people with responsibility Accountable is limited to intervening when a deadline is missed. Other forms of interaction, such as checking that an activity was correctly performed, are not allowed.

#### 3.1.3. Modelling software tools and BPMS

Modelling software tools, such as Visual Paradigm,^6^ facilitate the automatic generation of a RACI matrix from a resource-aware BPMN model. Specifically, the responsibility type Responsible can be automatically extracted and the RACI matrix can then be manually filled out to include information about the other types of responsibilities. However, the output is just used for documentation purposes, since BPMN does not support the definition of responsibilities Accountable, Consulted and Informed.

Signavio Process Editor^7^ also allows for defining RACI responsibilities in process models by making use of BPMN elements. While those models can be used for generating reports subsequently, process engines will not take into account the responsibilities Accountable, Consulted and Informed for automatic process execution.

The support for responsibility management is a novel functionality in BPMSs . Bizagi^8^ and ARIS (Scheer 2000) allow for the definition of RASCI responsibilities in BPMN models by making use of extended attributes in process activities. Nevertheless, similar to the tools focused on modelling, only the responsibility Responsible is considered for execution and the rest are used for process documentation and reporting. RACI matrices can be defined in the Red Hat JBoss BPM Suite^9^ aside of a process model for broader documentation of the responsibilities involved in the process (Cumberlidge 2007). To the best of our knowledge, only (YAWL) (Adams 2016) slightly supports responsibility-aware process execution by means of the concept of secondary resources (human and non-human), which may assist in the completion of the work (hence providing support). Any kind of support for responsibility modelling and execution other than Responsible is still missing, however, in other BPMSs, such as Camunda^10^ and Bonita BPM.^11^

#### 3.1.4. Research proposals

Due to the limitations of the process modelling notations and systems for responsibility management a few research proposals have been developed to support the assignment of different responsibilities to process activities and the automation of such responsibility-aware process models. In particular, Grosskopf (2007) extended BPMN 1.0 to support accountability.

Resource Assignment Language (RAL) (Cabanillas et al. 2015b; Cabanillas, Resinas, and Ruiz-Cortés 2011a) is an expressive language for defining resource assignments that supports all the creation patterns (Russell et al. 2005). RAL is independent of the process modelling notation. Therefore, it can be decoupled from the process model or it can be integrated in it, as shown in Cabanillas, Resinas, and Ruiz-Cortés (2011) with BPMN. Furthermore, RAL is suited to be used for modelling any kind of responsibility as long as that is supported by the process modelling notation with which it is used.

A graphical notation with a similar expressive power than RAL (RALph) was designed to allow for graphically defining resource assignments in process models (Cabanillas et al. 2015a). Similarly to the case of RAL, RALph is not actually equipped with support for modelling specific responsibilities. Therefore, that support depends on the process modelling notation with which RALph is used. Otherwise, the notation should be extended.

To a greater or lesser extent, these proposals only address responsibility modelling and they do not provide details about the implications on the execution of the responsibility-aware process models generated.

Since process execution is also a concern and the different responsibilities modelled with a process should also be considered at run time, the approach described in Cabanillas, Resinas, and Ruiz-Cortés (2011b) presented a pattern-based mechanism for including specific activities in a BPMN model that represent accountability, support, consultancy and notification functions. The result is thus a responsibility-aware executable process model that can be automated by BPMN process engines. However, due to the extra elements added in order to include RASCI responsibilities, the model is likely to become unreadable and deviate from the original one, hence turning out to be less eligible for other purposes, such as documentation, due to the large amount of implementation details. As an illustrative example, applying this technique to the scenario described in Section 2, the number of process activities would increase from 5 to 15. Moreover, the RASCI patterns defined are fixed and hence, there is no flexibility for adapting the joint use of the responsibilities to the organisational needs. Our preliminary work in this area (Cabanillas, Resinas, and Ruiz-Cortés 2012) also generated executable process models provided with RASCI information avoiding the aforementioned readability problem. However, flexibility remained an issue, as the way of including responsibilities in the process model was fixed.

### 3.2. Template-based process modelling

Process templates have been defined with different notations and used for different purposes and in different domains. For instance, BPMN templates were defined for generating so-called business process studios by means of model transformations (Mos and Cortés-Cornax 2016). Configurable processes rely on process fragments or templates for adapting an abstract process to a specific context. They have been used, e.g., for addressing the problem of variability in service implementation, a.k.a. service differentiation, with BPEL (Tao and Yang 2007) as well as the problem of reference model implementation with Configurable epc (C-EPC) (Recker et al. 2005). In addition, configurable processes have been applied in industry to solve real problems, as described in Gottschalk, van der Aalst, and Jansen-Vullers (2007) for SAP processes.

Most of these approaches, however, focus on control-flow aspects of business process and disregard other perspectives. Nevertheless, notations like the one presented in La Rosa et al. (2011) allow for defining configurable process models considering control flow, data and resources. These three perspectives are also supported by a template-based approach for modelling process performance indicators (del-Río-Ortega et al. 2016).

**Table 3.** Support for responsibility management in business processes. √ indicates that the feature is supported, ≈ indicates that the feature is partly supported, − indicates that the feature is not supported, and *n/a* indicates that the evaluation criterion is not applicable.

Group	Approach	Responsibilities	Modelling	Execution	Flexibility
Generic	RAM (Website 2016)	Any	out	–	n/a
BP modelling notations	BPMN 2.0 (OMG 2011)	Any	in	–	n/a
	BPMN 2.0 (OMG 2011) BPEL4People (OASIS 2009)/WSHumanTask (OASIS 2010)	RAI	in	✓	≈
Modelling tools and BPMSs	Visual Paradigm	RACI	out	–	n/a
	Signavio	RACI	in	–	n/a
	Bizagi	Any	in	–	n/a
	ARIS (Scheer 2000)	RASCI	in	–	n/a
	JBoss BPM Suite (Cumberlidge 2007) YAWL (Adams 2016)	RACI	out	–	n/a
	YAWL adams_yawl_2016	RS	in	✓	–
Research proposals	BPMN 1.0 Ext. by Grosskopf (2007)	RA	in	–	n/a
	RAL (Cabanillas et al. 2015b)	Any	in/out	–	n/a
	RALph (Cabanillas et al. 2015a)	Any	in	–	n/a
	RASCI patterns (Cabanillas, Resinas, and Ruiz-Cortés 2011b)	RASCI	in	✓	–
	RACI2BPMN (Cabanillas, Resinas, and Ruiz-Cortés 2012)	RASCI	out	✓	–
	*Our proposal*	Any	out	✓	✓

All the previous approaches have shown benefits for the purpose they were conceived. However, none of them has taken into consideration activity responsibilities since they did not specifically focus on the organisational perspective of business processes.

## 4. Motivation and research method

To conduct this research we have followed design science principles as suggested by Hevner et al. (2004) and, in particular, we have applied the *design science research methodology* (DSRM) (Peffers et al. 2007) as follows:

**Problem identification and motivation phase**: In this phase, we reviewed both research proposals related to our research question, i.e., responsibility management applied to executable business processes, and how this is currently applied in industry. Table 3 collects those that take into consideration more than one type of responsibility. Specifically, the following aspects are analysed:
  - The responsibilities supported, in most cases in terms of the well-known RASCI responsibilities (Website 2014).
  - The way in which responsibilities are modelled, differentiating between ‘in’ (i.e., the resource-related information is represented within the process model) and ‘out’ (i.e., the resource-related information is separated from the process model). This relates to the degree of decoupling.
  - The support for the automated execution of processes including the different responsibilities.

Several conclusions can be drawn from this analysis. First, many proposals from industry (notations, modelling tools and BPMS) recognise the importance of modelling different types of responsibilities for each activity and provide some kind of support for them, although this support is limited to modelling and documentation purposes, i.e., business-oriented process models.

Second, the RASCI concepts seem to be the most extended mechanism to model responsibility management in business processes. Indeed, many software modelling tools and BPMS use them and automatically generate RAMs to model or document responsibilities. The biggest limitation of this way of proceeding is that the expressiveness of RAMs for resource assignment in business processes is restricted to three creation patterns, namely, *Direct-based Distribution, Role-based Distribution and Organisational Distribution*. Patterns like SoD or BoD are not supported by default by RAMs . This is not a significant problem if RAMs are used solely for documentation purposes because they can be accompanied with a description of these concerns in natural language. However, it is an important limitation if RAMs were used as the model to guide the automated execution of a process in a BPMS.

Finally, the support for automating the resulting responsibility-aware process models is limited. When existing, it either does not cover all the RASCI responsibilities or is not flexible enough to accommodate different interaction patterns between the people that collaborate in an activity with different responsibilities.

The result is that there is a gap between the responsibility types that are modelled in business-oriented process models such as the one described in the running example (Section 2) or the use case (Section 7.3) and the ability of BPMSs to automate their execution. Consequently, to enforce their correct execution, it is necessary to implement them by manually adding activities for them in the executable process model. This is not desirable, especially when the process model has many activities (it is not uncommon to find process models with more than 15 activities (Mendling, Reijers, and van der Aalst 2010)) and there are several responsibilities for each activity (which is not uncommon either, e.g., in best practice frameworks). For instance, in our running example, despite its small size, at least 8 activities have to be added manually to model all responsibilities, one for each responsibility type assigned to an activity. The problem gets even worse when either the process or the responsibility assignment changes, because the modified process has to be changed by hand again. This involves first checking what has changed, then understanding the impact of this change in the modified process model and finally, changing the modified process model appropriately. This time-consuming and error-prone task is the problem we are facing in this research.

**Objective of the solution phase**: The objective defined in this phase was to bridge the gap between the responsibility management in business-oriented process models and executable process models by developing an approach that automates part of this manual process required to add different responsibilities to the executable process model. We also set two goals for such an approach, derived from the analysis depicted in Table 3:
  G1. Generalisability: in order to adapt to the organisational structure, the responsibility modelling technique should be able to deal with any kind of responsibility instead of sticking to a predefined set of them
  G2. Flexibility: in order to increase usability, the approach should leave freedom to each organisation to define how the interaction between the people that collaborate in an activity with different responsibilities takes place. Furthermore, the process flow chosen has a direct impact on process performance, as described in Lam, Ip, and Lau (2009).
**Design and development phase**: This phase involved the design and development of two novel artefacts, namely, (i) the RAM^BI^ metamodel, which extends RAMs with information required for process execution (cf. Section 5), and (ii) a template-based technique called RAM2BPMN for transforming such information into BPMN elements that can be interpreted by a BPMN process engine (cf. Section 6).
**Demonstration phase**: This phase involved the development of a software prototype that effectively showed that it was possible to transform the information of RAM^BI^ models into BPMN elements automatically using templates. Furthermore, it also showed that the solution is platform independent since it was used to generate process models that were executable in different BPMS (cf. Section 7.1).
**Evaluation phase**: The proposal has been evaluated in two different directions. On the one hand, we have used our approach to model interaction patterns between the people that collaborate in an activity with different responsibilities to validate that our solution was flexible enough to accommodate those interactions (cf. Section 7.2). On the other hand, we have applied our approach to two real scenarios in order to show its applicability and the advantages gained by its use (cf. Section 7.3).

These phases were developed by means of several iterative cycles. In an initial cycle, we partially defined the problem, reviewed existing research literature and current solutions from industry, and developed an initial solution that included a first version of the RAM^BI^ metamodel and a predefined interaction for RASCI responsibilities (Cabanillas, Resinas, and Ruiz-Cortés 2011b). Then, subsequent cycles were necessary to refine the RAM^BI^ metamodel, to make the approach independent of RASCI responsibilities and to give flexibility to the approach by means of the template-based technique.

## 5. RAM^BI^ : resource assignment matrices with binding information

A typical RAM enables an assignment of resources based on the organisational entities placed at the columns of the matrix, which usually are organisational roles. This limits the expressiveness of the resource assignments in two directions. On the one hand, it is not possible to set additional constraints related to the person assigned to the activity, such as requiring specific capabilities or excluding performers of previous activities to enforce an SOD . For instance, in the example of Section 2 the PhD student that is responsible for the activity Send Authorisation is not any PhD student but the one that prepared the authorisation form. On the other hand, it limits the ability to put additional constraints on the organisational entities used as columns. For instance, in the same example, the role project coordinator should not refer to any project coordinator, but the coordinator of the project to which the authorisation that is being requested belongs.

In the remainder of this section, we first introduce the RAM^BI^ metamodel, then we present its semantics and at last, we provide details of a specific instantiation of the metamodel with a specific resource assignment language.

### 5.1. RAM^BI^ metamodel

RAM^BI^ matrices (i.e., RAMs with binding information) is the extension we propose to overcome these limitations of the expressiveness of RAMs. It complements a RAMs with *binding information* that provides the specific conditions that the individuals have to fulfil in order to participate with a specific type of responsibility in an activity.

These conditions are expressed using a resource assignment language. From an abstract perspective, a resource assignment language L is composed of a set of expressions that use the different entities that are part of an organisational metamodel, such as people, roles or positions, for selecting elements of an organisational model defined according to the organisational metamodel. In other words, it can be seen as a query language for the organisational model. Accordingly, the semantics of a resource assignment language L can be defined by a function $map_{L}$ that maps each expression in L to a set of elements of the organisational model that is being queried.

**Figure 2.**
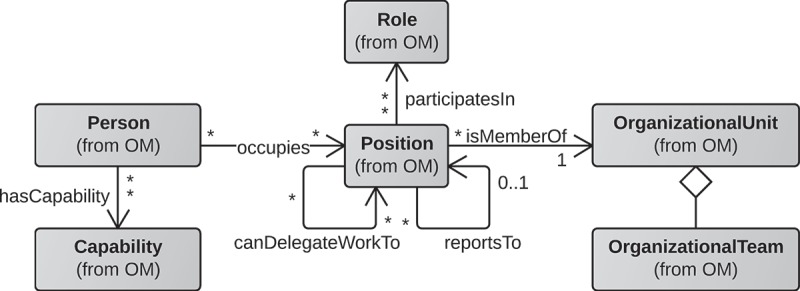
Excerpt of the organisational model described by Russell et al. (2004).

For instance, a resource assignment language L that queries an organisational model defined according to the organisational metamodel depicted in Figure 2 could have expressions that select people that play a certain role (e.g., selecting all people that have the role *researcher*) or that have performed a previous activity in the process (e.g., selecting all people that have performed activity Prepare authorisation in the current instance), amongst others. Furthermore, a resource assignment language also can have expressions to select not only people but other elements of an organisationl model such as organisational units or roles. For instance, we can query the model to obtain all positions that belong to an organisational unit.

Using a resource assignment language, a RAM^BI^ model can be formalised as follows.

Definition 1. *Let* TD *be the responsibility types that can be used in a RAMs, and let* L *be a resource assignment language where* κ *and* χ *are the set of expressions to select organisational entities and people, respectively. A RAM^BI^ is a tuple* M=(A,OR,BR,oc,ar), *where:*

- $A=\left\{a_{1},...,a_{n}\right\}$ is the set of activities that appear in the rows of the RAM^BI^, A≠∅.
- $OR=\left\{or_{1},...,or_{n}\right\}$ is the set of organisational roles that appear in the columns of the RAM^BI^, OR≠∅.
- BR⊆A×OR×TD is the set of *BoundRole*s defined in M. A *BoundRole* (a,or,td) represents a type of responsibility td assigned to a *Role* or of the organisational model for a given *Activity* a of a process. In other words, *BoundRoles* represent the letters that appear in the cells of a RAM.
- oc:BR→κ is a partial function that assigns an organisational context to a *BoundRole*.
- ar:BR→χ is a partial function that assigns additional restrictions to a *BoundRole*.

In this definition, the binding information is provided by functions oc and ar, namely: (i) oc indicates additional restrictions that apply to the organisational entity used as column, such as the organisational unit to which the role must belong for a specific *BoundRole*; and (ii) ar is used to specify restrictions on the people that can have a certain responsibility in an activity, e.g., to have knowledge on a specific subject or to have performed a previous activity in the process.

Table 4 defines a set of auxiliary functions that are used in the following sections to manipulate the RAM^BI^ matrix M.

**Table 4.** Auxiliary functions for the RAM^BI^ metamodel.

Function	Definition
$or_{M}:BR\to OR$	It obtains the organisational role used in a *BoundRole*. Formally: Let br=(a,or,td)∈BR be a *BoundRole* in M, $or_{M}(br)=or$.
$resps_{M}:A\to P(TD)$	It obtains the responsibility types used in any bound role of the given activity according to M. Formally: $resps_{M}(a)=\{t|\exists or\in OR((a,or,t)\in BR)\}$.
$filter_{M}:A\times TD\to P(BR)$	It obtains the bound roles that refer to the given activity and responsibility type according to M. Formally: $filter_{M}(a,t)=\{(a^{\prime },or^{\prime },t^{\prime })\in BR|a=a^{\prime }\wedge t=t^{\prime }\}$.
$hasTD_{M}:A\times TD\to \left\{true,false\right\}$	It evaluates to true if and only if the given activity has at least one organisational role that has the given responsibility type assigned in M. Formally: $hasTD_{M}(a,t)\Leftrightarrow t\in resps_{M}(a)$.
$onlyTD_{M}:A\times TD\to \left\{true,false\right\}$	It evaluates to true if and only if the given responsibility type is the only responsibility type defined for the given activity according to M. Formally: $onlyTD_{M}(a,t)\Leftrightarrow \left\{t\right\}=resps_{M}(a)$.
$anyTD_{M}:A\to \left\{true,false\right\}$	It evaluates to true if and only if the given activity has at least one organisational role that has one responsibility type assigned in M. Formally: $anyTD_{M}(a)\Leftrightarrow resps_{M}(a)\neq \emptyset$.

### 5.2. Semantics of the RAM^BI^ metamodel

Giving semantics to a RAM^BI^ means to determine which are the people that can have a certain type of responsibility in a given activity according to the information included in a RAM^BI^ model. To do so, we rely on a function $map_{BR}$ that determines the people that fulfill the conditions of a *BoundedRole*. Intuitively, this function can be defined as follows:

1. If the *BoundedRole* b has no binding information (i.e., oc(b) and ar(b) are undefined), then the people that fulfill the condition of b are those people that play the role identified in the *BoundedRole* (e.g., any researcher).
2. If the *BoundedRole* b has an organisational context defined (e.g., a reserch project), then the people that fulfill the condition of b are those people that play the role identified in the *BoundedRole* within the organisational context provided (e.g., any researcher in a specific research project.)
3. If the *BoundedRole* b has an additional restriction defined (e.g., being responsible for activity Prepare authorisation), then the people that fulfill the condition of b are those people that play the role identified in the *BoundedRole* and fulfill the additional restrictions (e.g., researchers that are responsible for activity Prepare authorisation.)
4. Finally, if the *BoundedRole* b has both an organisational context and an additional restriction defined, then the people that fulfill the condition of b are those that play the role identified in the *BoundedRole* within the organisational context provided and fulfill the additional restrictions.

This can be formalised as follows.

Definition 2. *Let* O *be an organisational model with* P *people. Let* L *be a resource expression language that can query model* O *whose semantics is defined by function* $map_{L}$. *Let* OC *be the set of all possible organisational contexts for a role* or∈OR *in model* O, *and let* p(or,oc)⊆P *be the set of people that have role* or *in context* oc *according to* O. *The semantics of each BoundRole of a RAM^BI^* M=(A,OR,BR,oc,ar) *is defined by function* $map_{BR}:BR\to P(P)$ *as follows:*

$$map_{BR}(b)=\{\begin{array}{cc} {⋃}_{c\in OC}p(or_{M}(b),c) & \;\;\;\;if\;oc(b),\;ar(b)\;undef.\\ {⋃}_{c\in map_{L}(oc(b))}p(or_{M}(b),c) & \;\;\;\;if\;oc(b)\;def.,ar(b)\;undef.\\ {⋃}_{c\in OC}p(or_{M}(b),c)\cap map_{L}(ar(b)) & \;\;\;\;if\;oc(b)\;undef.,ar(b)\;def.\\ {⋃}_{c\in map_{L}(oc(b))}p(or_{M}(b),c)\cap map_{L}(ar(b)) & if\;oc(b)\;def.,ar(b)\;def. \end{array}$$

This function gives semantics to the resource assignment specified in each *BoundRole* (i.e., in each cell of the RAM^BI^). However, this does not specify the semantics when there are several *BoundRoles* with the same responsibility for the same activity (i.e., the same responsibility appears in more than one cell in the same row), which is common for responsibilities like Consulted, Support or Informed. Since no formal semantics for RAMs have been defined, several different interpretations can be done in different contexts:

1. Only one person can do the responsibility. This person must fulfil the conditions of the resource assignment specified in *any* of the bound roles defined for the same responsibility and the same activity.
2. Only one person can do the responsibility but this person must fulfil the conditions of the resource assignment specified in *all* the bound roles defined for the same responsibility and the same activity.
3. Several people can do the responsibility; one for each bound role defined for the same responsibility and the same activity. Each of these people must fulfil the conditions of the resource assignment specified for their respective bound role.

These interpretations can be formalised as follows.

Definition 3. *Let* M=(A,OR,BR,oc,ar) *be a RAM^BI^ model, and let* TD *be the type of responsibilities that can be used in* M:

1. The semantics of the first interpretation is defined as function $orMap_{M}:A\times TD\to P(P)$ as follows: $orMap_{M}(a,td)=\cup_{br\in filter_{M}(a,td)}map_{BR}(br)$
2. The semantics of the second interpretation is defined as function $andMap_{M}:A\times TD\to P(P)$ as follows: $andMap_{M}(a,td)=\cap_{br\in filter_{M}(a,td)}map_{BR}(br)$
3. The semantics of the third interpretation is defined as function $map_{M}:A\times TD\to P(P(P))$ as follows: $map_{M}(a,td)=\{map_{BR}(br)|br\in filter_{M}(a,td)\}$

The additional constraints included by binding information may cause undesirable side effects if there is no person in the organisational model that meets all the constraints. This could happen, for instance, if we set as additional constraint for the clerk that is responsible for activity Prepare authorisation in the running example that she has the capability of speaking English and it turns out that there is no clerk with such a capability in the project. The identification of these situations is called consistency checking and it has been studied in detail in the literature (Cabanillas et al. 2015b). Specifically, a resource assignment, such as the one specified by a RAM^BI^ model, is consistent if it is always possible to find a potential participant for an activity during any execution of the process for any responsibility type that appears in the resource assignment.

Since inconsistencies are caused by the constraints included by binding information specified in a resource assignment language, the ability to check the consistency of a RAM^BI^ model is directly related to the ability to check the consistency of the resource assignment language used in its binding information. This is possible with some resource assignment languages such as RAL (Cabanillas et al. 2015b). In the next section we introduce RAL and detail how consistency checking can be implemented with it.

### 5.3. Using RAM^BI^ with RAL

As discussed in the previous sections, a RAM^BI^ model relies on an external resource assignment language to define its binding information. Next, we illustrate how RAM^BI^ can be used with RAL (Cabanillas et al. 2015b). We have chosen RAL because of its high expressive power, capable of supporting all the creation patterns (Cabanillas, Resinas, and Ruiz-Cortés 2011) and because it has a well-defined semantics that enable the implementation of analysis operations such as consistency checking. However, other resource assignment languages could also be used.

RAL is a modular (DSL) that specifies a set of *expressions* and *constraints* to define resource assignment conditions independently of any specific process modelling notation. It is composed of five modules:

- *RAL Core* allows defining generic resource assignment expressions based on resource’s characteristics. For instance, it allows assigning an activity to one of two specific resources with the expression (IS Betty) OR (IS Anna).
- *RAL Org* extends RAL Core and allows selecting people according to their organisational information based on an organisational metamodel that involves persons, capabilities, positions, roles and organisational units (cf. Figure 2). RAL Org expressions and constraints were defined to cover all of the relations that appear in the metamodel, among others, such that:
  - The *occupies* relation is supported by *PositionConstraint*, e.g., HAS POSITION AssistantProfessor.
  - The *isMemberOf* relation is supported by *UnitConstraint*, e.g., HAS UNIT InstituteForIB.
  - The *participatesIn* relation is supported by *RoleConstraint*, e.g., HAS ROLE Researcher, or HAS ROLE Researcher IN UNIT InstituteForIB.
  - The *hasCapability* relation is supported by *CapabilityConstraint*, e.g., HAS CAPABILITY PhDdegree.
- *RAL Data* and *RAL DataOrg* allow selecting individuals, positions, roles or organisational units indicated in a data field of a data object of a process, according to the BPMN (OMG 2011) specification of the business process data perspective.^12^ For instance, the following expression specifies that the resource allowed to execute an activity is indicated in the data field *Applicant* of the data object *Application*: IS PERSON IN DATA FIELD Application.Applicant.
- *RAL AC* stands for RAL Access-Control and it extends RAL Core to enable the specification of access-control constraints, such as bod (e.g., IS ANY PERSON responsible for ACTIVITY SubmitPaper) and sod (e.g., NOT(IS ANY PERSON responsible for ACTIVITY FillForm)).

The use of a specific resource assignment language in a RAM^BI^ model has an impact concerning the type of binding information that can be used in it. Specifically, for RAL it involves the following aspects:

1. RAL has two expressions to select people according to roles, namely: HAS ROLE r and HAS ROLE r IN UNIT u. Therefore, the organisational context that can be given in RAL to an organisational role is always the organisational unit in which the role is played (e.g., the role Coordinator can be played in a department or a project.) Consequently, expression κ refers to an organisational unit and p(or,c) can be resolved using RAL expression HAS ROLE or in UNIT c.
2. All previously described RAL resource assignment expressions can be used to add additional constraints to a *BoundedRole*. This means that all the creation patterns (Cabanillas, Resinas, and Ruiz-Cortés 2011) can be used to select people in RAM^BI^ models. For instance, it is possible to add additional constraints related to the capabilities of the resource usingRAL Org expression HAS CAPABILITY capabilityID or based on the responsibilities it takes in other activities using aRAL AC expression such as IS ANY PERSON responsible for ACTIVITY a.

**Figure 3.**
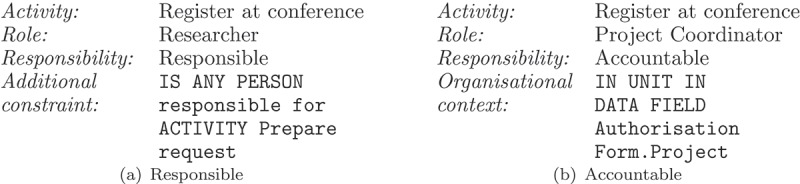
RAM-BI models and RAL expressions for activity Prepare Authorisation.

Figure 3 illustrates in a simplified way the use of the RAM^BI^ metamodel with RAL using as example activity *Register at Conference* of the scenario described in Section 2 and Table 2. As depicted in Figure 3(a), the person with responsibility type Responsible is the researcher who prepared the authorisation request. Hence, there is a bod access-control constraint defined with RAL AC. As for responsibility type Consulted, it is assigned to the coordinator of the project that funds the trip to the conference, indicated in the data object *Authorisation form*. Therefore, the organisational context is defined with aRAL DataOrg expression.

Finally, the well-defined semantics of RAL enables the implementation of several analysis operations such as consistency checking (Cabanillas et al. 2015b). Therefore, to check the consistency of a RAM^BI^ model that uses RAL as its language for binding information, we just need to translate a RAM^BI^ model into a RAL expressions and then leverage the consistency checking operation that has been implemented for RAL . This translation can be easily done using RAL expressions HAS ROLE or for the case when oc(b) and ar(b) are undefined, HAS ROLE or IN UNIT u for the case when oc(b) is defined and the operator AND to compose expressions for the case when ar(b) is defined. The result is function $map_{BR}^{RAL}:BR\to RALExpr$ that can be defined as follows:

$$map_{BR}^{RAL}(b)=\{\begin{array}{cc} HAS\;ROLE\;or_{M}(b) & if\;oc(b),\;ar(b)\;undef.\\ HAS\;ROLE\;or_{M}(b)\;IN\;UNIT\;oc(b) & if\;oc(b)\;def.,ar(b)\;undef.\\ (HAS\;ROLE\;or_{M}(b))\;AND\;(ar(b)) & if\;oc(b)\;undef.,ar(b)\;def.\\ (HAS\;ROLE\;or_{M}(b)\;IN\;UNIT\;oc(b))\;AND\;(ar(b)) & if\;oc(b)\;def.,ar(b)\;def. \end{array}$$

Finally, $orMap_{M}^{RAL}$ and $andMap_{M}^{RAL}$, both can be straightforwardly defined by joining the RAL expressions obtained by $map_{BR}^{RAL}$ for each bound role defined for the same task duty and the same activity with OR and AND, respectively.

## 6. RAM2BPMN : using RAM^BI^ with BPMN models

**Figure 4.**
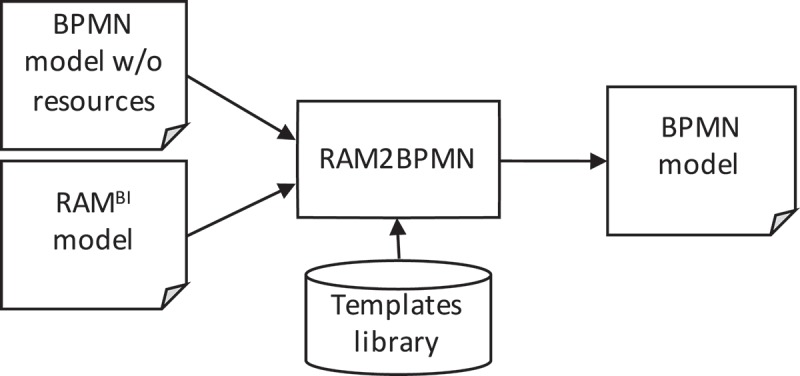
Overview of RAM2BPMN.

RAM2BPMN is our approach to enable current BPMS to execute BPMN processes in which people with different responsibilities collaborate to complete process activities. An overview of RAM2BPMN is depicted in Figure 4. The core idea is to take a BPMN model without resource-related information and a RAM^BI^ model as inputs and to automatically generate a new BPMN model in which the only responsibility defined for each activity is Responsible, but which includes new activities to model the semantics conveyed by the other responsibilities included in the RAM^BI^ model. More specifically, RAM2BPMN turns every activity for which some type of responsibility different than Responsible is defined into a subprocess. We refer to the subprocesses created during the transformation as *RAM subprocesses*. A RAMs subprocess is a regular BPMN subprocess that includes the specific tasks for all the responsibilities that people may have during the execution of the activity of the original process. RAMs subprocesses are created from *collaboration templates*. A collaboration template specifies how people with different responsibilities interact with each other to carry out an activity of a process. The collaboration template used is chosen at design-time amongst a library of templates depending on the specific requirements of the activity.

We have opted for this approach for several reasons. First, because it ensures that RAM2BPMN can generate models for any BPMS because the generated model only relies on defining the resource responsible for each activity, which is the only responsibility supported in BPMN by default. Second, because the additional complexity that results from including the information about different responsibilities is found only inside the RAMs subprocesses and hence, it does not affect the overall understandability of the initial process. Furthermore, the only difference between the resulting model and the initial one from the visualisation standpoint is that tasks are transformed into collapsed subprocesses.

**Figure 5.**
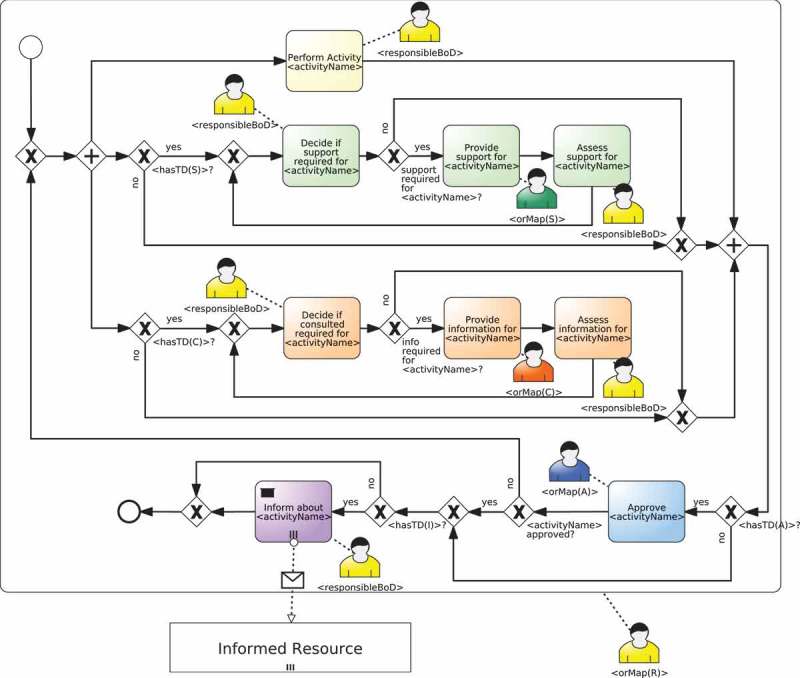
Example of a template that models the interaction of RASCI responsibilities as it was introduced in in Cabanillas, Resinas, and Ruiz-Cortés (2011b).

Figure 5 depicts an example of template^13^ that models the interaction of the RASCI responsibilities as it was introduced in Cabanillas, Resinas, and Ruiz-Cortés (2011b). This interaction establishes that the approval action (Accountable) takes place after the completion of the work developed for the activity (Responsible), and only then the notification action (Informed) can be performed. There is also a loop to redo the work in case it does not get the approval by the resource with the responsibility Accountable. Actions of responsibility Consulted and responsibility Support are considered to take place in parallel with the task performed by the resource with responsibility Responsible. Finally, the template includes two decision activities performed by the resource with responsibility Responsible to decide whether support or consultation are required. The template also has some placeholders that have to be filled with information that comes from the RAM^BI^ model and the definition of the activity in the BPMN model.

Another relevant aspect is that not all possible RAM^BI^ models can be used with the template depicted in Figure 5. In particular, it requires that there is *exactly one bound role for responsibility responsible* and *at most one bound role for responsibility accountable* for each activity, whereas there can be any number of bound roles for the other responsibilities. In general, each template may require a specific cardinality for the responsibilities of a RAM^BI^ model.

**Figure 6.**
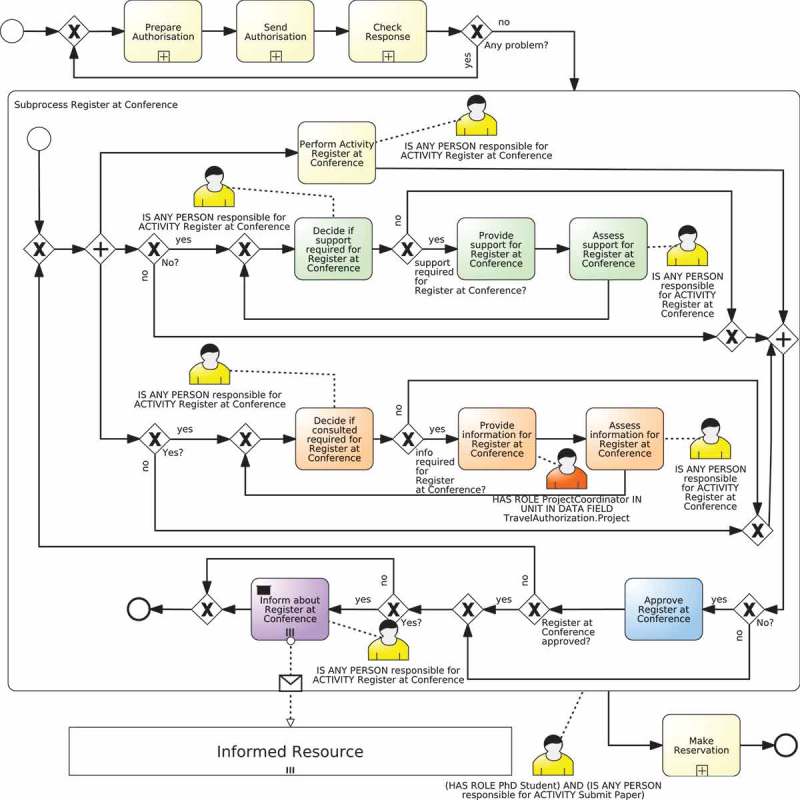
Example of the instantiation of static template in Figure 5 for activity Register at Conference of the process defined in Figure 1.

The procedure that creates a RAM subprocess from a template for a specific activity of the process is the *instantiation of a template*. This procedure uses the information of the RAM^BI^ and the BPMN models to fill the placeholders of the template with activity-specific information and to compose the RAMs subprocess, when necessary. For instance, Figure 6 depicts the result of the instantiation of the template in Figure 5.

The advantage of using templates is that each organisation may define their own library of templates to specify how people involved in an activity of a process with different responsibilities interact with each other, thus providing flexibility on the way these interactions are carried out. Furthermore, although one template is usually used for all activities of a process or even several processes, it may be the case that for a specific activity (or process) one might be interested in using a different template. For instance, for a time-sensitive activity, one might be interested in a template in which the person accountable for the activity does not only supervise the outcome of the activity but also the completion time. This can be done, e.g., by designing a template in which the task performed by the person responsible for the activity has a timer that triggers a supervision task performed by the person accountable for the activity when a predefined time has passed. Section 7.2 discusses some examples of different templates that can be used with RASCI responsibilities.

The support for different templates for each activity in the process is modelled by means of function templ that links each activity to the template that must be used:

Definition 4. **(Assignment of templates to activities)** *Let* A *be the set of activities of a process and* T *be the set of all possible templates, function* templ:A→T *specifies which template is used for each activity of the process.*

Algorithm 1. RAM2BPMN: Including resource assignment information from RAM^BI^ into a BPMN model

1: **IN**: *BPMN; M* = (A;OR;BR;oc;ar); *templ*

2: **Pre**: A ⊆ activities of process *BPMN*

3: **Pre**: The resource assignment of *M* is consistent with BPMN

4: **Pre**: *compatible(M; templ)*

5: **OUT**: *BPMN’* model with resource information

6: *BPMN*’ ← *BPMN*

7: **for all** activity a in the business process *BPMN* do

8:  **if** *anyTD(a)* **then**

9:   *T ← templ(a)*

10:   *subprocess_a_ ←* instantiation of template *T* using *BPMN* and *M*

11:   replace in *BPMN’* activity a by *subprocess_a_*

12:  **end if**

13: **end fo**r

14: return **BPMN’**

Based on the concept of template and its instantiation, the RAM2BPMN algorithm can be formalised as detailed in Algorithm 6. The input is a resource-unaware BPMN model, i.e., a model without resource assignments, (bpmn); a RAM^BI^ model (M), and a templ function.

The algorithm has three preconditions, namely: the activities of the RAM^BI^ model must be a subset of the activities in the BPMN model (specifically, those that cannot be automated); the resource assignment of M must be consistent with bpmn (cf. Section 5.2), and the templates chosen by the templ function must be compatible with the RAM^BI^ model in terms of cardinality (cf. Section 6.1).

The algorithm can be outlined as follows. First, the bpmn model is cloned into $bpmn^{\prime }$ (line 6). Then, the following three steps are executed for each activity of the process (line 6) that has a resource assignment in the RAM^BI^ model (anyTD(a)) (line 6). First, the template associated to the activity is selected (line 6). Second, the template is instantiated using the resource assignment information provided by the RAM^BI^ model (line 6). Finally, the activity of $bpmn^{\prime }$ is replaced by a subprocess created using the template instantiated in the previous step (line 6). The last step of the algorithm is to return the new $bpmn^{\prime }$ in which activities have been replaced by RAMs subprocesses (line 6.

Next, we delve into the two parts of the algorithm that require more details, namely: the concept of compatibility between RAM^BI^ model and template (line 6), and the definition and instantiation of templates (line 6). The consistency checking has already been discussed in Section 5.2.

### 6.1. Cardinality of templates

As discussed before, not all RAM^BI^ models can be used with a specific template because templates may require a specific cardinality for the responsibilities of the RAM^BI^ model (e.g., they may limit the number of people with responsibility Responsible to exactly one). The cardinality of a template is defined with the template together with the specification of the interaction it models, such as the one in Figure 5, and can be formalised as follows.

Definition 5. **(Cardinality of a template)** *Let* T *be a template and let* TD *be the set of responsibilities supported by* T. *The cardinality of each responsibility is defined by means of function* $card_{T}:TD\to IN^{0}\times (IN^{0}\cup^{\left\{n\right\}})$ *such that* $card_{T}(td)=(x,y)$ *means that the template requires at least* x *bound roles and at most* y *bound roles for a responsibility* t∈TD, *such that* x≤y. *If* y=n, *then any number of bound roles greater than or equal to* x *is allowed.*

For convenience, for each $card_{T}(td)=(x,y)$ we define functions $min_{T}(td)=x$ and $max_{T}(td)=y$ to represent the minimum and maximum number of bound roles for a responsibility t∈TD.

Based on the cardinality of a template, the compatibility of a template and a RAM^BI^ model for an activity can be defined. Intuitively, a template is compatible with a RAM^BI^ model for an activity A if all responsibilities specified in the RAM^BI^ model for A fulfil the cardinality restrictions specified in the template. This can be formalised as follows:

Definition 6. **(Compatibility of a template)** *Let* M=(A,OR,BR,oc,ar) *be a RAM^BI^ model*, $TD^{M}$ *be the set of responsibilities used in* M, card *be the cardinality function of a template* T, *and* $TD^{T}$ *be the set of responsibilities used in* T. *The template* T *is compatible with the RAM^BI^ model* M *for an activity* a∈A *iff the number of bound roles specified in* M *related to activity* a *for each responsibility is within the constraints specified by the cardinality function* card:

$$\begin{array}{cc} compatible(M,T,a)\Leftrightarrow \forall td\in resps_{M}(a) & (td\in TD^{T}\wedge \\ & (min_{T}(td)\leq \left|filter_{M}(a,td)\right|\wedge \\ & max_{T}(td)\neq n\Rightarrow \left|filter_{M}(a,td)\right|\leq max_{T}(td))) \end{array}$$

Finally, equipped with this definition, it is easy to define the compatibility of an assignment of templates to activities (templ) with a RAM^BI^ model as follows.

Definition 7. **(Compatibility of an assignment of templates)** *Let* M=(A,OR,BR,oc,ar) *be a RAM^BI^ model and* templ *an assignment of templates to activities. The assignment of templates to activities* templ *is compatible with* M *if and only if it assigns a template that is compatible with* M *for each activity in* A:

$$\begin{array}{cc} compatible(M,templ)\Leftrightarrow \forall a\in A & (templ(a)\;is\;defined\wedge \\ & compatible(M,templ(a),a)) \end{array}$$

### 6.2. Definition and instantiation of templates

Figure 5 depicts an example of template for RASCI responsibilities. However, this shows just one possible way in which a template can be defined and instantiated. As a matter of fact, the only requirement imposed by the RAM2BPMN algorithm is that the result of the instantiation should be a subprocess configured using information from the BPMN and the RAM^BI^ models. This means that different approaches can be used to define templates and their choice depends on the characteristics of the template that is being modelled as discussed in Section 6.2.3.

In the remainder of this section, we detail two different approaches for the definition of collaboration templates and discuss their advantages and drawbacks, although other approaches could also be designed. To illustrate them, we use the aforementioned interaction of RASCI responsibilities introduced in Cabanillas, Resinas, and Ruiz-Cortés (2011b).

#### 6.2.1. Static templates

A static template is a process model defined in BPMN (cf. Figure 5) that details the interaction between people with different responsibilities within an activity with just two peculiarities:

- Placeholders are used in the resource assignments and the names of the activities that will be replaced with values obtained from the RAM^BI^ and/or the BPMN models during instantiation.
- XOR gateways are included in the process for enabling or disabling the activities specific to a responsibility. To this end, placeholders can also be used in the conditions of the gateways.

The instantiation mechanism of these static templates just involves the replacement of all the placeholders that appear in the template. This is done by iterating over all these placeholders and replacing their value by one obtained from the RAM^BI^ or the BPMN model. Table 5 depicts a list of the placeholders that can be used in the static template and their replacement.

**Table 5.** Frequent placeholders and their replacement.

Placeholder name	Replacement of the placeholder
<activityName<	Name of the activity obtained from the BPMN model
<responsibleBoD>	RAL expression IS ANY PERSON responsible for ACTIVITY X, where *X* is the activity for which the RAMs subprocess is created.
<f>	Where *f* is $resps_{M}$, or $anyTD_{M}$. It represents the value of the function according to the RAM^BI^ model for the activity for which the RAMs subprocess is created.
<f(TD)>	Where f is $filter_{M}$, $hasTD_{M}$, $onlyTD_{M}$, $map_{M}^{RAL}$, $orMap_{M}^{RAL}$, or $andMap_{M}^{RAL}$. It represents the value of the function according to the RAM^BI^ model.
<f(BR)>	Where *f* is $or_{M}$, or $map_{BR}^{RAL}$. It represents the value of the function according to the RAM^BI^ model.

Figure 5 depicts an example of a static template and Figure 6 depicts its instantiation. The template models the interaction of RASCI responsibilities introduced in Cabanillas, Resinas, and Ruiz-Cortés (2011b) and includes gateways to bypass parts of the process in case the responsibility is not assigned for the activity. The template also has placeholders for the name of the activities so that they include the name of the activity for which the RAM subprocess is created, and the resource assignments (expressed using BPMN annotations in the diagram). In particular, activities *Provide support, Provide information* and *Approve activity* are assigned to the responsibilities Support, Consulted and Accountable, respectively. The other activities, which include the two decision and the two assessment activities, are performed by the responsibility Responsible. In the latter case, the RAL expression IS ANY PERSON responsible for ACTIVITY T is used. This allows every element within the subprocess to make reference to the performer of the activity (i.e., the new subprocess), since according to BPMN (OMG 2011) the allocation related to a subprocess is made before starting the activities that compose it. Note also that the responsibility Informed is different from the others because it refers to the *target* of the notification action (i.e., the people that are informed), not to the *holder* of the responsibility like in the rest of cases. Furthermore, given the definition of the responsibility Informed (cf. Section 3.1.1), it is reasonable to consider it an external participant of the process because, independently of her responsibilities with respect to other process activities, for the activity in question she is a target and not an executor. For this reason, the activities that correspond to the responsibility Informed are modelled as *send tasks* (OMG 2011).

#### 6.2.2. Fragment-based templates

Static templates provide a simple yet useful mechanism to define templates. However, more complex mechanisms can be devised. One source of inspiration for these mechanisms are configurable business processes (La Rosa et al. 2011). A configurable process model captures a family of related process models in a single artifact. Such models are intended to be configured to fit the requirements of specific organisations or projects, leading to individualised process models (van der Aalst et al. 2010). Therefore, templates can be seen as a type of configurable business processes and their instantiation can be seen as a configuration of them (Kumar and Yao 2012). Consequently, most approaches for defining and setting up configurable business processes can be adapted for defining collaboration templates.

In particular, fragment-based templates are based on the approach proposed in Kumar and Yao (2012) for designing flexible process variants using templates and rules. The templates are made up of two different elements that can change from template to template, namely: a set of process fragments, at least one for each responsibility; and a composition algorithm that is used for putting together all those fragments and for enabling or disabling the tasks specific to a responsibility.

**Figure 7.**
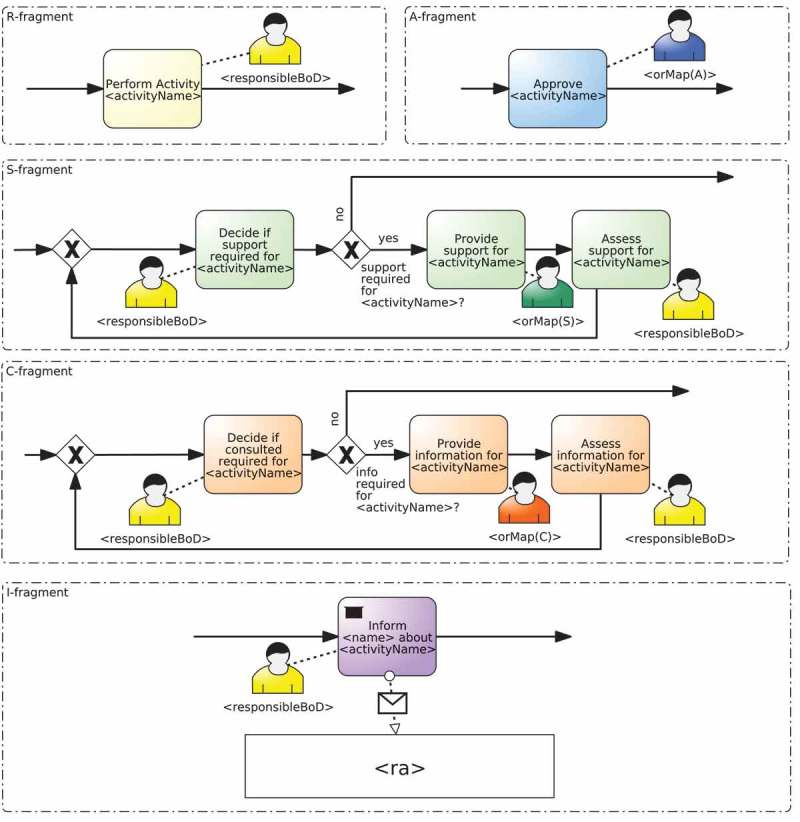
Example of the fragments defined for each of the RASCI responsibilities in a fragment-based template.

Fragments must be subprocess graphs with single entry and single exit nodes (also denoted as *hammocks* in graph literature (Weber, Reichert, and Rinderle-Ma 2008)) that represent the tasks that are necessary to carry out a given responsibility. Not only the same placeholders as with static templates but also ad-hoc placeholders can be used in the fragments. The value for these placeholders must be provided by the composition algorithm. Figure 7 depicts an example of fragments that belong to a fragment-based template (more examples can be found in Section 7.2). Specifically, five process fragments named R-fragment, A-fragment, S-fragment, C-fragment, and I-fragment are defined, one for each RASCI responsibility. Each of them have their corresponding placeholders in the names of the activities and the resource assignments.

Concerning the composition algorithm, the idea is to combine the accompanying process fragments in a meaningful way based on the information provided by the RAM^BI^ model and the BPMN model. To describe how the process fragments are composed together, we suggest the use of change patterns (Weber, Reichert, and Rinderle-Ma 2008). Change patterns and in particular, a type of them called adaptation patterns, allow users to structurally modify a process model using change operations defined in terms of the process model (e.g., adding an activity in parallel to another one) instead of change primitives defined in terms of the underlying graph (e.g., adding a single node, two control flow edges, and the connectors between them) (Weber, Reichert, and Rinderle-Ma 2008). A consequence of this is that change operations provide a compact way of defining changes on a process because each change operation involves one or more change primitives (e.g., adding an activity in parallel involves up to seven change primitives). Furthermore, these changes tend to be easier to understand because they are defined in a higher-level way. All these reasons make change operations a suitable language to define these compositions. Table 6 shows a summary of the most frequent change operations.

**Table 6.** Frequent change operations for the implementation of fragment-based templates.

Change operations	Meaning
insertSequential(F1, F2)	Inserts the fragment F2 right after F1
insertParallel(F1, F2)	Inserts the fragment F2 in parallel with the fragment F1
embedInLoop(F, loop, exit)	Embeds the fragment F in a loop. loop and exit are the loop condition and the exit condition, respectively

Together with change operations, placeholders defined in Table 5 can also be used as variables and functions in the composition algorithm. The placeholders of the process fragments are replaced during the execution of the composition algorithm. This is done by means of function *replace*, which receives the fragment with placeholders and returns the same fragment with all placeholders replaced with their corresponding value. The values for ad-hoc placeholders must be provided as parameters of function *replace*.

**Figure 8.**
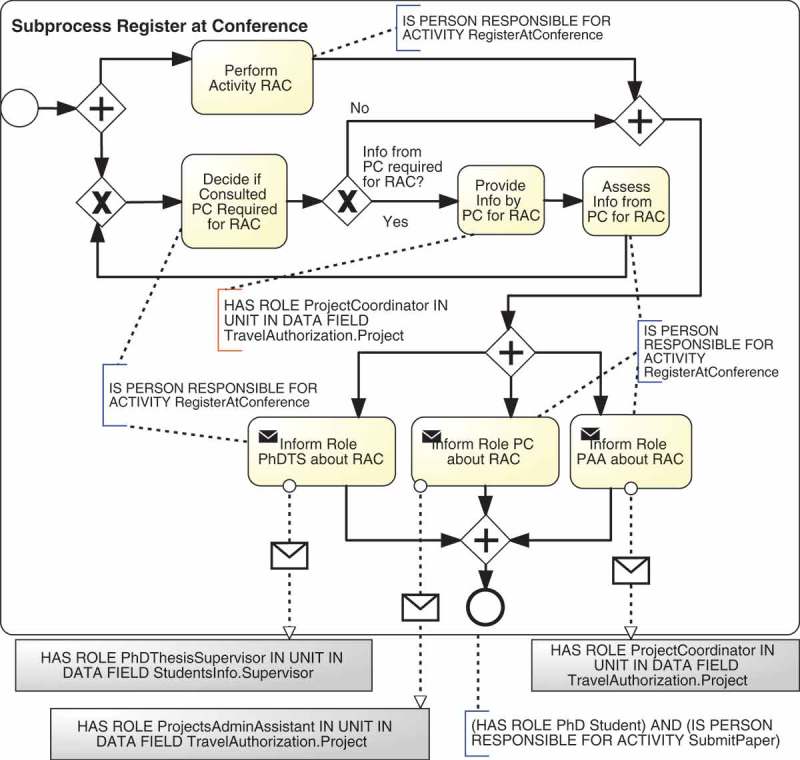
Instantiation of the template depicted in Figure 2 for activity *Register at Conference.*

Algorithm 2 shows an example of composition algorithm of a fragment-based template that composes the fragments defined in Figure 7. This algorithm is executed as part of the instantiation of the fragment-based template it belongs to, which means that it would be invoked within line 10 of Algorithm 1. The algorithm starts by replacing the placeholders in the fragment for the responsibility Responsible (line 8). Then, if responsibility types support or consulted have been defined in the RAM^BI^ model (lines 5 and 8) it inserts in parallel fragments for the responsibilities Support and Consulted (lines 6 and 9, respectively). Next, if responsibility type accountable has been defined in the RAM^BI^ model (line 11), it inserts sequentially the fragment for the responsibility Accountable (line 12) and embeds the whole process in a loop based on the decision made by the person accountable for the activity, i.e., whether the work is approved or not (line 13). Finally, if responsibility type informed has been defined in the RAM^BI^ model (line 15) it inserts a fragment for the responsibility Informed for each organisational role that must be informed. This is done in several steps. It iterates over all organisational roles with responsibility Informed defined in the RAM^BI^ model for the given activity (line 17). For each of them, it obtains its resource assignment and name (lines 18 and 19) and it inserts in parallel fragments for each of them in a temporal variable informFragment (lines 20–23). Finally, it inserts sequentially all these parallel fragments contained in informFragment (line 26). Figure 8 depicts the instantiation obtained after executing the composition algorithm for activity Register at conference according to the RAM^BI^ model depicted in Table 2.

Algorithm 2. Example of the composition algorithm of a fragment-based template that support RASCI responsibilities

1: **IN**: $TD=\left\{R;A;S;C;I\right\}$ are the RASCI responsibilities supported by this template.

2: **IN**: *R* – *fragment; S* – *fragment;C* – *fragment;A* – *fragment; I* – *fragment* are the fragments defined in Figure 7.

3: **OUT**: composed fragment

4: base ← replace(R-fragment)

5: **if** hasTD(S) **then**

6:  base ← insertParallel(base, replace(S-fragment))

7: **end if**

8: **if** hasTD(C) then

9:  base ← insertParallel(base, replace(C-fragment))

10: **end if**

11: **if** hasTD(A) then

12:  base ← insertSequential(base, replace(A-fragment))

13:  base ← embedInLoop(base, not approved, approved)

14: **end if**

15: **if** hasTD(I) then

16:  informFragment ← empty fragment

17:  **for all** br ∈ *filter(I)* **do**

18:   *ra ← map_M_(br)*

19:   name ← orM(br)

20:   **if** informFragment = empty fragment **then**

21:    informFragment ← replace(I-fragment, ra, name)

22:   **else**

23:    informFragment ← insertParallel(informFragment, replace(I-fragment, ra, name))

24:   **end if**

25:  **end for**

26:  base ← insertSequential(base, informFragment)

27: **end if**

28: **return** base

#### 6.2.3. Discussion

The decision on which template mechanism to use depends on the requirements of the specific scenario in which they are being applied. Fragment-based templates enable reusing fragments in different templates. So, one can have a repository of different fragments for the same responsibility and combine them in different ways with different composition algorithms. In addition, fragment-based templates allow a more flexible definition since the composition algorithm provides a more fine-grained control of the results of the instantiation. For instance, in Figure 8 several inform fragments were added, one for each organisational role with the responsibility Informed in the RAM^BI^ model, which is something that cannot be easily done with static templates. Finally, the subprocess obtained from fragment-based templates tends to be easier to understand since it only includes the responsibilities that have been assigned to the activity.

However, static templates are easier to build since they do not need a composition algorithm and hence, it is not necessary to deal with its implementation. Moreover, in fragment-based templates, it is harder to detect deadlocks or livelocks introduced during the instantiation because the composition algorithm may change the control flow depending on which responsibilities are enabled for the activity at hand. This does not happen in static templates, in which the control flow is the same all the time.

Therefore, the conclusion is that fragment-based templates should be used in cases in which we are interested in building templates that have many commonalities or in cases in which we require a more fine-grained control of the instantiation. Otherwise, static templates are more appealing since that approach avoids the complexity of developing the composition algorithm.

## 7. Validation

We have validated the feasibility of the approach with a reference implementation, its flexibility with a repository of templates for responsibility modelling on the basis of the RASCI responsibilities, and its applicability by using the approach with a real scenarion from the railway automation domain.

### 7.1. Implementation

**Figure 9.**
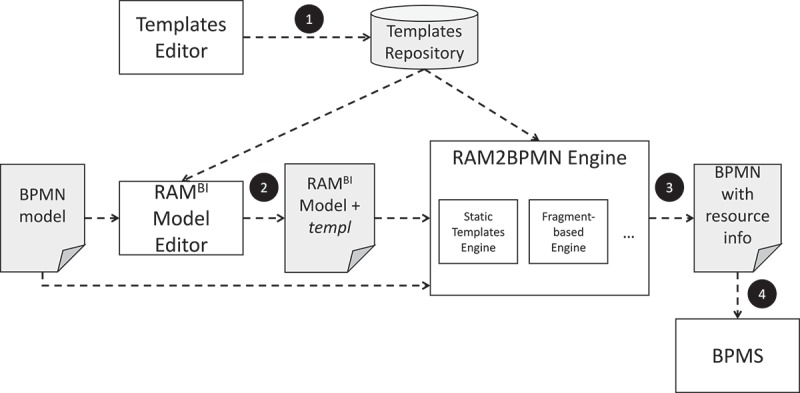
Overview of the architecture implement to support the RAM2BPMN procedure.

We have developed a reference implementation of the RAM2BPMN algorithm as well as an editor for RAM^BI^ models and a repository of templates. Furthermore, we have also implemented templates according to the modelling alternatives for the RASCI responsibilities described in Section 7.2. An overview of the architecture designed to support the RAM2BPMN procedure is depicted in Figure 9. A use case for the whole architecture is described as follows:

1. A template designer builds a set of templates using a template editor and stores them in the template repository.
2. A RAM^BI^ model is defined for each BPMN model whose resource assignment we want to extend. To this end, a process designer uses the RAM^BI^
								 Model Editor to define the RAM^BI^ for the given BPMN model. Furthermore, she also chooses the template that should be applied to each activity from amongst the templates stored in the template repository.
3. The RAM2BPMN engine uses the RAM^BI^ model, the BPMN model and the templates from the template repository to obtain a BPMN with resource information.
4. The BPMN model obtained in the previous step is deployed into a BPMS for its execution.

The templates used by the architecture can be either static templates or fragment-based templates. Furthermore, the architecture has been designed so that new types of templates could be easily added to the system.

All of the components that make up this architecture have been integrated into Collection of Resource-centrIc Supporting Tools And Languages (CRISTAL) (Cabanillas et al. 2012), a tool suite that provides support for the specification and automated analysis of the business process resource perspective. Information about the system can be found at 
						www.isa.us.es/cristal
					.

#### 7.1.1. Templates editor

In our current implementation, there is no dedicated template editor. Instead, a BPMN model editor is used to model the static templates or the process fragments for the fragment-based templates and then, they are manually stored in the templates repository.

#### 7.1.2. Templates repository

The templates repository is a Java library that stores the templates that have been designed for the organisation. Each template is composed of a template description file, which details the name of the template and its cardinality (cf. Section 6), and a set of companion files that are specific of each type of template. For instance, in a static template there is only one companion file, which is the BPMN model of the template; and in a fragment-based template there is one companion file for each process fragment and another one for describing the composition algorithm. The current implementation of the templates repository relies on a file-based storage for both template descriptions and companion files.

#### 7.1.3. RAM^BI^ model editor

**Figure 10.**
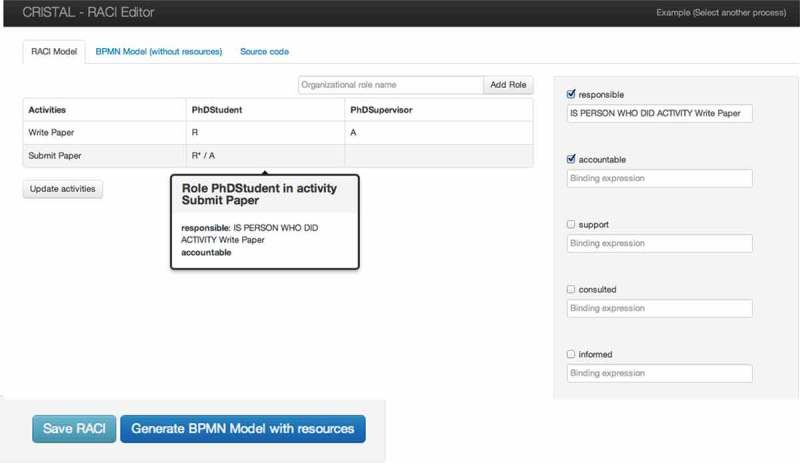
Screenshot of the RAM^BI^ Model Editor.

It is a Web application developed in Java that allows the user to define the resource information associated with the activities of a BPMN model by filling in a RASCI matrix (Website 2014) and optionally adding binding information with RAL (Cabanillas et al. 2015b), as shown in Figure 10. This editor also allows the user to specify the template that should be used for each activity in the process, i.e., function templ. Both the RAM^BI^ model and the function templ are serialised into the JSON file format.

#### 7.1.4. RAM2BPMN engine

The RAM2BPMN Engine implements the RAM2BPMN algorithm (cf. Algorithm 1). Specifically, it receives an XML file with the representation of a (resource-unaware) BPMN model, a JSON file with the RAM^BI^ model and the function templ, as well as a templates repository; and it returns the XML representation of the BPMN model with the RASCI information embedded in it. This functionality can be invoked from the user interface of the RAM^BI^ Editor (cf. Figure 10) or from the command line.

The RAM2BPMN Engine supports the two types of templates described in Section 6. Furthermore, the engine has been designed to allow new types of templates provided that the corresponding instantiation mechanism is integrated into the engine.

The resulting BPMN model generated is standard BPMN . Therefore, it can be manipulated in any process modelling tool and executed in a BPMN -compliant BPMS *that has the required support for the resource assignment language used in the RAM^BI^ model*.

### 7.2. Modelling alternatives for RASCI responsibilities

One of the main advantages of our approach is that it does not enforce any specific behaviour for the people with different responsibilities that work together on an activity, but it allows the organisation to model the interaction that best suits its requirements. Furthermore, a library of templates can be created by the organisation so that they can be reused in different processes.

In the following we validate this flexibility by modelling alternatives that can be considered for RASCI responsibilities. We focus on RASCI responsibilities because they are a well-known responsibility set used in many different domains. The modelling alternatives are obtained from our experience in different projects in which RASCI responsibilities have been modelled as well as from patterns identified in both the industry (Effektif 2016; OASIS 2010) and the related research literature (Brambilla, Fraternali, and Vaca 2011; Barchetti et al. 2012). Because the requirements of each organisation may lead to different alternatives, this is by no means a complete catalogue, but rather an illustration of the wide variety of possibilities that our approach enables.

To avoid confusion, in this section we use *activity* to refer to the activity of the original business process for which a RAMs subprocess shall be created and we use *task* to refer to each of the activities that are part of the RAMs subprocess. The modelling alternatives are introduced using BPMN 2.0 modelling concepts (OMG 2011).

#### 7.2.1. Responsible

This responsibility represents the execution of the activity itself and the coordination of other responsibilities involved in the execution of the activity such as Support, Consult or Informed. Therefore, the modelling alternatives for this responsibility come from the role it plays in the interaction with the other ones. Since these alternatives are also related to the other responsibilities, they are described in the section that discusses the respective modelling alternatives.

#### 7.2.2. Accountable

The modelling alternatives of this responsibility are based on whether the accountability refers to the quality of the work performed or it also refers to the fact that the work is finished in a timely manner. The former is the typical interpretation of accountability whereas the latter is useful for time-sensitive activities.

If accountability refers to the quality of the work performed, it usually involves an explicit approval of the work previously performed by the person responsible for the activity. This implies adding a task for that purpose in the template. If the work is not approved, then a loop is in place so that the person responsible for the activity has to do it again. An example of this can be found in the task *Approve Activity* < *activityName* > of the template of Figure 5. This behaviour has been identified as the Document Approval pattern in Effektif (2016)

If accountability refers to making sure the work is finished in time, it involves the inclusion of a trigger in the process that notifies the person accountable for the activity that it has not been completed in a predefined amount of time. From a modelling perspective, this involves adding a time-based non-interrupting boundary event to the task performed by the person responsible for the activity such that if a certain time has passed and the task has not been completed, the person allocated to the responsibility Accountable is notified. This behaviour has been identified as the Timed Escalation pattern in Effektif (2016) and it is also the way accountability is dealt used in OASIS (2010).

Both alternatives are not exclusive and can be used together in the same template.

#### 7.2.3. Support and consulted

**Figure 11.**
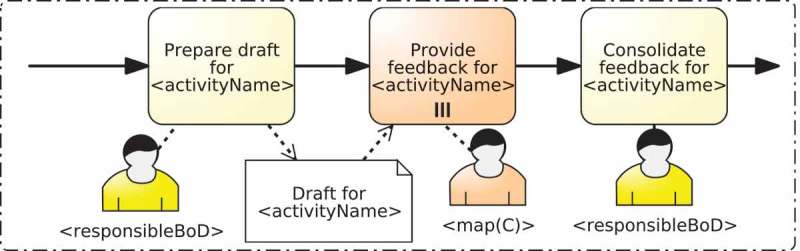
Template fragment that shows a modelling example for consult responsibility modelled as mandatory, done after some work, batches, group-based, system decided and not assessed.
**The moment in which support or consultation takes place**. There are two options: it can be done in parallel to the main task, or it can be done after some initial work is done. From a modelling perspective, the former case can be modelled as in Figure 5 in which support and consultation tasks are performed in parallel with task *Perform activity*. The latter case usually involves three stages: a first stage in which the person responsible for the activity performs a preliminary task; a second stage in which support or consultation is required; and a third stage in which the person responsible for the activity merges the contributions received. This general scheme is followed by several patterns identified in the literature. For instance, in the Collaborative Editing pattern described in Barchetti et al. (2012), the person responsible for the activity first creates a draft and then this draft receives contributions from all the people with the Support responsibility for the activity. Finally, the person responsible for the activity collects all contributions and finishes the activity. The same pattern but for the Consulted responsibility is described in Effektif 2016) as the Multiple Stakeholder Input pattern. This is the pattern depicted in Figure 11.
**The mechanism to request additional support or consultation**. In this case, there are two options: it can be required in batches, i.e., no additional support or consultation can be required before the current ones are done; or it can be required at any time. From a modelling perspective, the former case can be modelled as in Figure 5 or Figure 11. Instead, the latter case requires a mechanism to decouple the provision of support from the request so that different requests can be done regardless of whether the provision of support has finished. A way to do that is to make the provision of support a non-interrupting event-based subprocess that is executed when a signal is thrown. This is illustrated in Figure 12.

The responsibilities Support and Consulted share some modelling alternatives because they have a very similar nature. Both of them involve a person to collaborate with the person responsible for the activity in its execution, being their degree of involvement in it the only difference between them. There is a wide variety of modelling alternatives for these two responsibilities based on several characteristics of the interaction, namely:

**The optionality of the interaction**. There are two options: the participation of people with the Support or Consulted responsibility is mandatory, or their participation is optional. From a modelling perspective, the former case involves the execution of the task that provides the support or consultation as shown in Figure 11 with task *Provide feedback for* < *activityName* >. The latter case usually translates into the inclusion of a task prior to the task that provides the support or consultation in order to decide whether help is required. An example is task *Decide if support required for* < *activityName* > of the template of Figure 4, which takes place before the task *Provide support for* < *activityName* >. The decision task is usually performed by the person responsible for the activity, but it could also be performed by the person that has the Support or Consulted responsibility.

**Figure 12.**
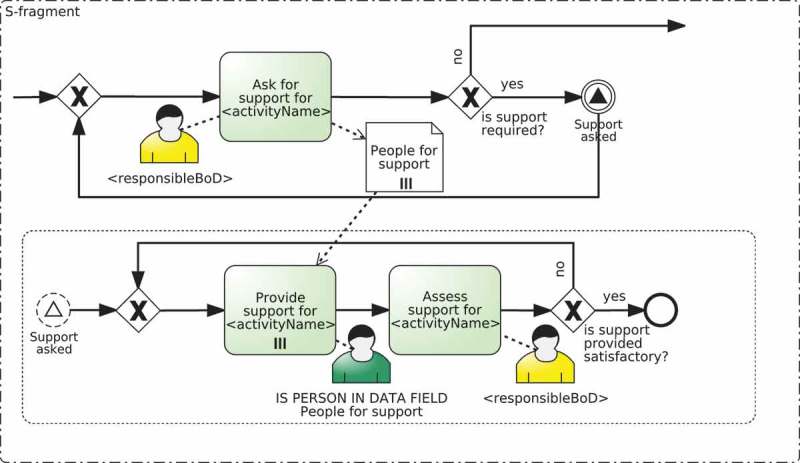
Template fragment that shows a modelling example for support responsibility modelled as optional, done in parallel, anytime, group-based, responsible decided and assessed.
**The number of people that can provide support or be consulted**. In this case, the support or consultation can be asked to one person at a time or to a group of people at the same time. Modelling the former is straightforward and an example is depicted in Figure 5. The latter can be modelled using either several parallel tasks or a multi-instance task. Using several parallel tasks is more appropriate if we want to control exactly how many people can provide support or consultation in parallel. It is also useful to have a higher degree of control concerning who decides who takes the responsibility (see next characteristic). Instead, if we are interested in providing freedom of choice at run time, multi-instance tasks are more convenient because all their parametrisation in terms of the number of parallel instances and conditions for cancellation are defined using run-time process data. Figures 11 and 12 depict this alternative.
**The decision on who performs the Support or Consulted responsibility**. A RASCI matrix defines a set of candidates to provide support or consultation for a given activity. However, it does not mean that all of them have to provide it. Someone has to choose the specific people that shall be allocated to the responsibility. There are typically two options to do so: either the person responsible for the activity explicitly chooses the specific people from amongst all candidates, or the choice is done following the mechanisms provided by the BPMS. The former option requires having a task performed by the person responsible for the activity in which she chooses the people she wants for support or consultation. This choice is stored in a data object that is used to assign these people to the tasks that represent the support or consultation by means of RAL expression IS PERSON IN DATA FIELD x. An example is depicted in Figure 12. This behaviour has been identified as the Required Role Assignment pattern in Effektif (2016). The latter option just requires using the RAL expression of the RASCI matrix assignment of the responsibility for the activity in the task that represents the responsibility. This is the way it is done in the template of Figures 5 and 11.
**The explicit assessment of support or consultation**. If support or consultation is explicitly assessed, then a task performed by the person responsible for the activity should be added after the task that represents the support or consultation. An example of this is depicted in Figures 5 and 12 by means of task *Assess support for* < *activityName* >.

In addition to the aforementioned characteristics, it is also possible to model the support tasks in a more structured manner. For instance, one may create a template in which the support follows a divide-and-conquer approach. In this case, the person responsible for the activity may decompose the work to be done in small work items and assign each of them to people that provide support to the activity. After all the contributions have finished, the person responsible for the activity can merge all the work items into a final result. This pattern is typically used in crowdsourcing scenarios (Kittur et al. 2011).

#### 7.2.4. Informed

This responsibility is modelled as a task that notifies the people that have to be informed about the state of the activity. Its modelling alternatives are based on the following characteristics of the interaction and a version of them are also present in the support WS-HumanTask (OASIS 2010) provides to responsibility informed:

**The moment at which people are informed**. The most typical behaviour is that people are notified at the end of the activity, i.e., after it has been performed and approved. This is how it is done in the example of Figure 5. However, other moments for notification can be included in the template, such as when the work related to the activity has been performed and it is waiting for approval. One may also want to notify the state of the activity after a certain amount of time has passed. This can be modelled by attaching a notification task to a time-based non-interrupting boundary event placed in the task representing responsibility Responsible. Note that all these alternatives are not mutually exclusive and hence, they can be used altogether in the template.
**The person in charge of informing**. The Informed responsibility is peculiar in the sense that the people assigned in the RASCI matrix are not the ones who perform the activity, but those who receive the notification. This means that someone has to perform the action of informing. One option is that the notification is sent automatically by the BPMS, e.g., as an email. If, on the contrary, the notification has to be sent by a person, that person might be either the person responsible for the activity or a person that provides support for the activity, since sending notifications can be seen as a supportive task.
**The way notifications are realised**. The *how* is usually domain-specific and may cover a wide set of options that range from an email to a web service invocation or a telephone call, among others. Furthermore, how people are informed is strongly related to who performs the notification because some notification mechanisms are easier to automate than others. For instance, a notification that involves sending an email or an SMS is easier to automate than making a phone call.

### 7.3. Use case

**Figure 13.**
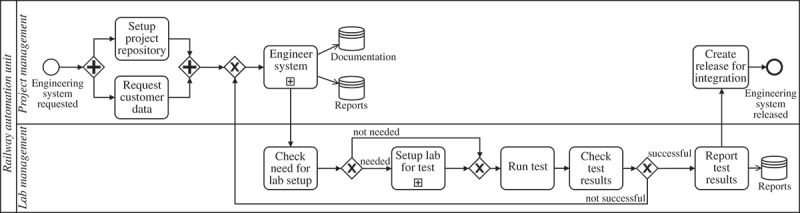
An engineering process in the railway domain.

We have applied our approch to a real scenario from industry in the railway automation domain, specifically to the process for releasing a new engineering system for a railway customer. The BPMN model of the process is depicted in Figure 13.

The process starts when a new agreement with a client has been signed by the project management team. A new repository is then created for the customer data by a technician and, at the same time, possible additional data are requested from the client by the engineer project manager. The next step is the actual engineering of the system, a subprocess in which the new system is built. It thus involves a large variety of resources and data coming from different data sources. The engineering project manager orchestrates and monitors the engineering tasks. Once the system is built, it must be tested before it is released for its use. That procedure takes place in a laboratory and comprises two steps: the test setup and the test execution. Prior to that, the testing project manager has to check whether the lab spaces needed must be set up for the test. Specific information about the lab settings and the system developed might be required in order to make such a decision. The employees of the organisation involved in the test setup and run steps are specialised in the execution of specific testing phases for specific types of systems, i.e., there may be an engineer who can perform the setup for a system $S_{1}$ and the test execution for a system $S_{2}$, another engineer who cannot be involved in the testing of system $S_{1}$ but can perform both activities for the system $S_{2}$, and so on. The setup tasks usually require one lab assistant working on one unit for a specific type of hardware in the laboratory; and the run activity usually requires several employees for its execution, in particular, one engineer who is responsible for conducting the tests and one lab assistant who provides support. When the testing of the system is finished, the testing project manager is notified, as they account for this activity. They then check the results of the test. If the results are not satisfactory, the system must be re-engineered. Otherwise, the testing project manager writes a final report that is archived together with the information generated containing the description of the test cases, test data, test results, and the outline of the findings. Information from the engineers involved in the building of the system may be necessary for writing such a report. Once ready, the engineer project manager is informed and proceeds to deploy an already complete and tested version of the engineering system, most likely helped by an engineer. The system integration team will later undertake the installation of the product, which constitutes a different process.

**Figure 14.**
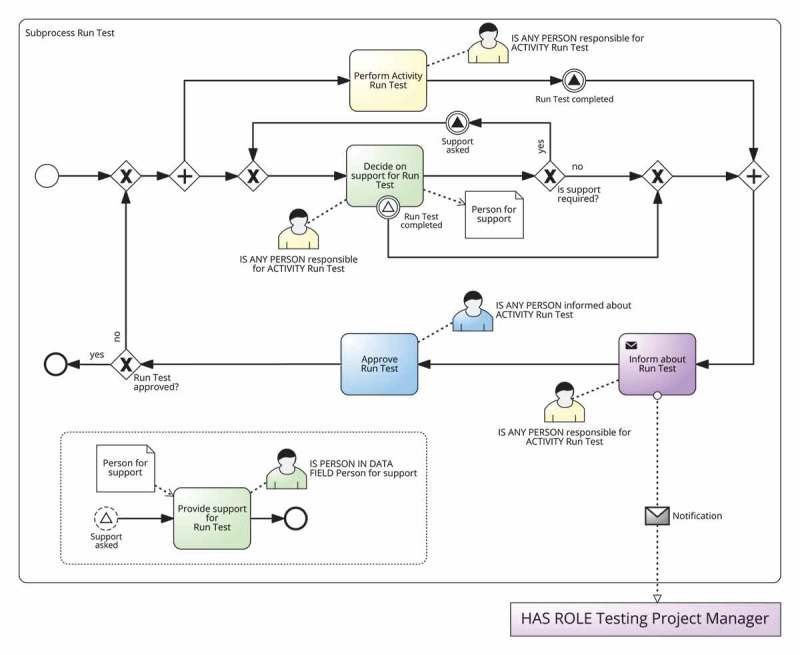
Composition of template fragments for activity Run Test.

Table 7 shows the RASCI matrix of the process excluding the two subprocesses and Figure 14 illustrates a template that models the interaction of RASCI responsibilities for activity *Run test*. The template is done by composing template fragments for the responsibilities involved. Note that the alternatives described in Section 7.2 must be considered for this purpose. In particular, regarding support, it is optional for the activity and, in case it is required, it can be requested at any time and will be carried out in parallel with the rest of the execution performed by the person responsible for the activity, i.e., the engineer in charge. Only one lab assistant supports the engineer in running the test. Assessment by the engineer is not necessary as the work will later be checked for approval. In order for that to happen, the testing project manager is informed about the completion of the work and proceeds to check the outcome, hence following the Document Approval pattern.

**Table 7.** RASCI matrix for the engineering system release process.

		Engineer	Testing	Lab	Lab	
	Engineer	Manager	Manager	Manager	Assistant	Technician
Setup project		A				R
repository						
Request		R				
customer data						
Check need	C		R	C		
for lab setup						
Run	R		A, I		S	
test						
Check			R	I		
test results						
Report	C	I	R			
test results						
Create release	S	R				
for integration

## 8. Discussion

From the analysis of the research literature and the current approach followed in industry to responsibility management in business processes, it becomes apparent that there is an increasing demand and support for modelling activities that involve several people with different responsibilities. However, as far as process automation is concerned, the only responsibility that is managed during process execution in most proposals is the one related to carrying out the work required for the completion of the activity. Moreover, in the few cases that they support other types of responsibilities, this support is very limited in terms of generalisability and flexibility as discussed in Section 4. This is a problem because the other responsibilities have to be implemented by manually adding activities for them in the executable process model, which is a time-consuming and error-prone task, specially for processes with a large number of activities or frequent changes in the resource assignment.

This paper introduces two novel artefacts, namely the RAM^BI^ metamodel and the RAM2BPMN algorithm that contribute to improve the existing support for responsibility management in executable business processes in order to alleviate the aforementioned problem. Specifically, these two artefacts together enable the execution, in current BPMN engines, of business processes in which several people participate in the same activity with different responsibilities. This is done thanks to RAM2BPMN that automatically extends business process models to include in them the information about the people with different responsibilities that are specified in a RAM^BI^ model. This extension is done leveraging templates that can be defined by the user and specify how the interaction between the people with different responsibilities must be carried out. Although the use of templates to transform BPMN models has been done elsewhere, this approach is the first time in which templates have been used to deal with the organisational perspective of business processes. Specifically, by using them, our approach fulfils the two goals defined in Section 4, which are not supported by any of the proposals analysed in Section 3, namely:

1. It is generic in terms of the types of responsibilities that can be used in it because it does not impose a predefined set of responsibilities, but it can use the most convenient set for each situation.
2. It is flexible in the sense that the interaction between the people that collaborate in an activity with different responsibilities is not predefined, but can be adapted to specific scenarios. Furthermore, our proposal enables the creation of libraries of templates that allows reusing different interaction patterns across activities, processes, and even organisations.

Furthermore, the way RAM2BPMN is designed provides the following additional advantages:

1. It is platform independent in the sense that the models obtained can be used by any BPMS that supports BPMN .
2. It is transparent in the sense that, although it transforms the original process model into a new one, the new elements are always embedded into subprocesses, which means that the original structure of the process model is unaltered. This is an advantage for the monitoring of the process and the understandability of audit logs because, although the information provided by the BPMS refers to the extended process instead of the original one, since the process structure is unaltered, it is straightforward to translate the information to the original process.

It is also remarkable that although we use BPMN as the process modelling language, the same ideas can be applied to other process modelling languages by adapting the details of the approaches described in this paper to their features. Similarly, where we use RAL one could use another language for defining resource assignments. In this case, the overall expressiveness would be that of the resource assignment language used.

However, our proposal has two main limitations. First, in the current approach, the template has to deal with the errors that occur during the process, e.g. a person that should be informed is not informed because the email server does not work. Although this is enough for most cases, it limits the ability to reuse error recovery strategies in different templates. One possible solution could be to extend RAM2BPMN so that the designer can specify which recovery strategy must be use in each activity independently of the template chosen. Second, since we are using RAL to define the resource assignments, mechanisms to process RAL expressions are necessary in order to be able to automatically calculate the potential holders of the responsibilities at run time; however, this support can be easily integrated into BPMS, as described in Cabanillas et al. (2015b).

## 9. Conclusions and future work

In this paper we have presented an approach to extend the existing support for responsibility management in business processes. This approach is based on a metamodel and a technique to enable the execution, in current BPMN engines, of business processes in which several people participate in the same activity with different responsibilities. A prototype of the approach has been implemented, and it has been evaluated by modelling existing interaction patterns between people that collaborate in an activity with different responsibilities and by applying it to two real scenarios.

From this work we conclude that the existing support for responsibility management can be improved in several directions. On the one hand, our proposal of RAM^BI^ models shows how modelling responsibility assignments can be decoupled from process models unlike what is usually done in languages such as BPMN that put together the information concerning control flow and human resources. This enables a separation of concerns between process behaviour and resource assignment, which provides a better visualisation of the information concerning resource assignment. This is especially useful when modelling complex processes or when the designs of the control flow and the resource assignments are done by different persons.

On the other hand, this work shows that it is possible to create a library templates that model different interactions patterns between people that participate in the same activity with different responsibilities. What is interesting is that these templates are reused across different activities and even different processes. This encourages a new research line focused on identifying these interaction patterns and determining in which situation they are useful. Some preliminary work already exists coming from industry (Effektif 2016) and academy (Brambilla, Fraternali, and Vaca 2011; Barchetti et al. 2012).

As next steps, we plan to explore additional use cases together with industry. In particular, we are interested in coming up with a set of interaction patterns that can be found in different cases and to define them using a similar approach to the design patterns in software engineering (Gamma et al. 1993). In addition, we also plan to extend our prototype implementation to integrate it into an open source BPMS such as Camunda.^14^ Specifically the idea is to integrate the implementation as a plugin so that a model is transformed right after being deployed in the BPMS in a way that is transparent to the user. We think that this would contribute to the dissemination of the tool and its integration by startups or third party companies.

## Notes

1. Please notice that the values for roles and persons are fictitious.

2. In BPMN a process takes place within a single pool. Diagrams with two or more pools, in which messages between the pools are exchanged, are called collaborations.

3.
http://www.sixsigmaonline.org/six-sigma-training-certification-information/articles/the-efficient-use-of-a-six-sigma-raci-matrix.html.

4.
http://en.it-processmaps.com/products/itil-raci-matrix.html.

5. For the sake of simplicity, the organisational roles that are not involved have been omitted.

6.
http://www.visual-paradigm.com/tutorials/racichart.jsp.

7.
http://academic.signavio.com/help/en/responsibility_assignment_acco.htm.

8.
http://www.bizagi.com/.

9.
http://www.jboss.org/products/bpmsuite/overview/.

10.
https://camunda.com/.

11.
http://www.bonitasoft.com/.

12. A *Business Process* can have a set of *Data Objects*, which can contain one or more *Data Fields*, whose value may change throughout process execution.

13. From now on, when we use the term *template*, we refer to *collaboration templates*.

14.
http://camunda.org.
